# Supplementary figures and images for: A Prognostic Model Using Post-Steroid Neutrophil-Lymphocyte Ratio Predicts Overall Survival in Primary Central Nervous System Lymphoma
Source: Cancers (Basel). 2022 Apr 3;14(7):1818. doi: 10.3390/cancers14071818 (PMC8997514; doi:10.3390/cancers14071818)

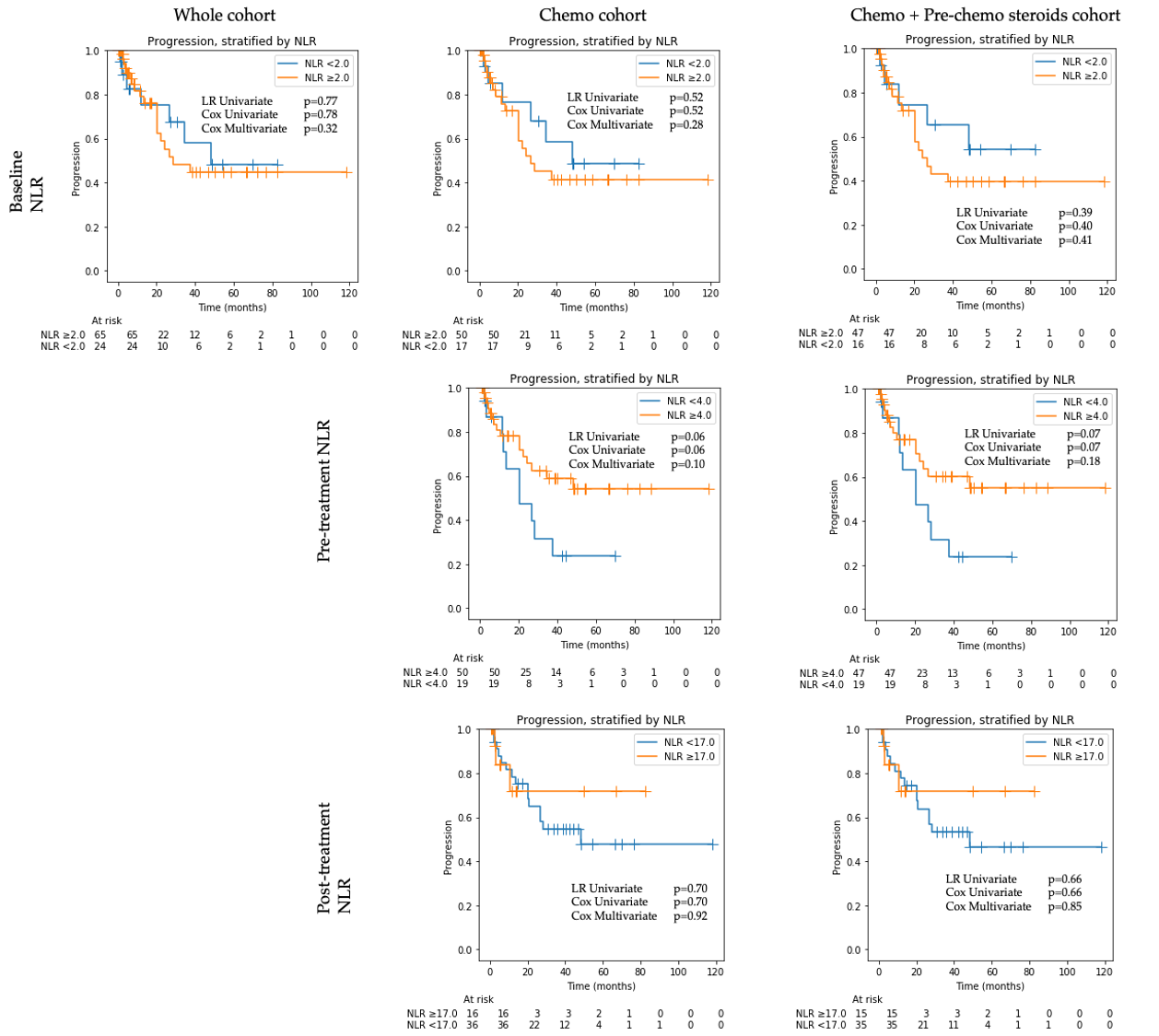

Supplement: Supplementary file 1 [file cancers-14-01818-s001.zip › supp fig 1.png]

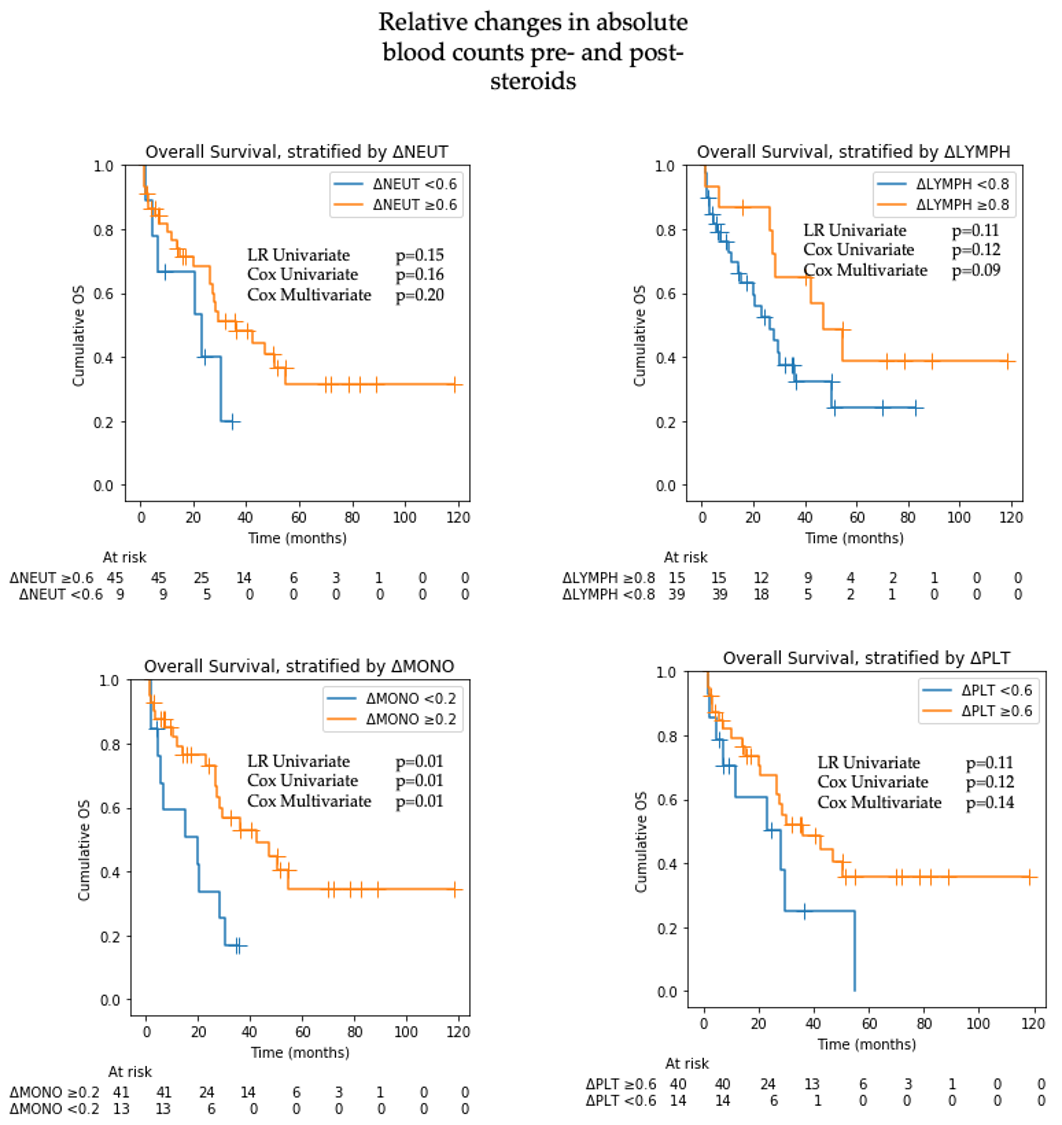

Supplement: Supplementary file 1 [file cancers-14-01818-s001.zip › supp fig 2.png]

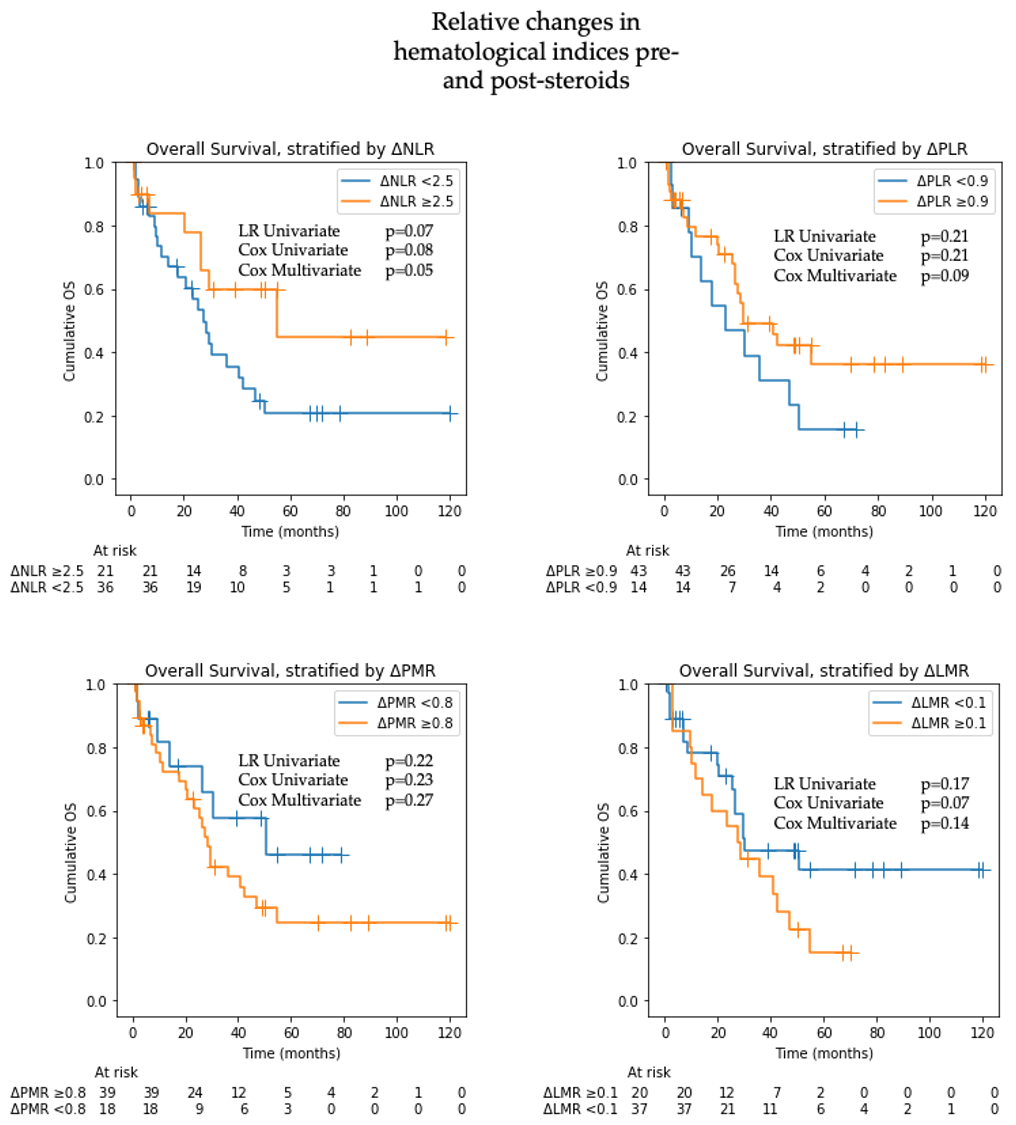

Supplement: Supplementary file 1 [file cancers-14-01818-s001.zip › supp fig 3.png]

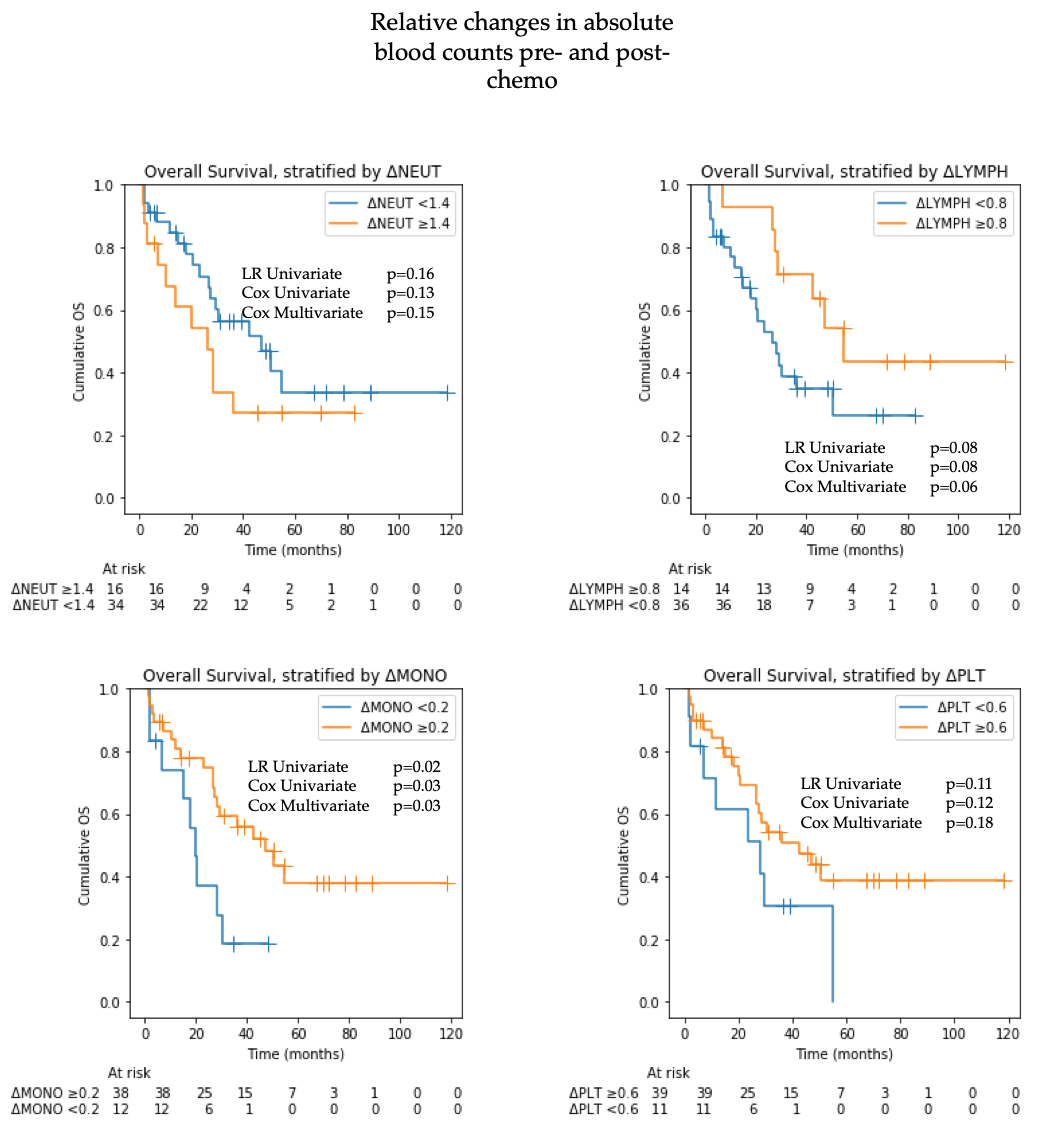

Supplement: Supplementary file 1 [file cancers-14-01818-s001.zip › supp fig 4.png]

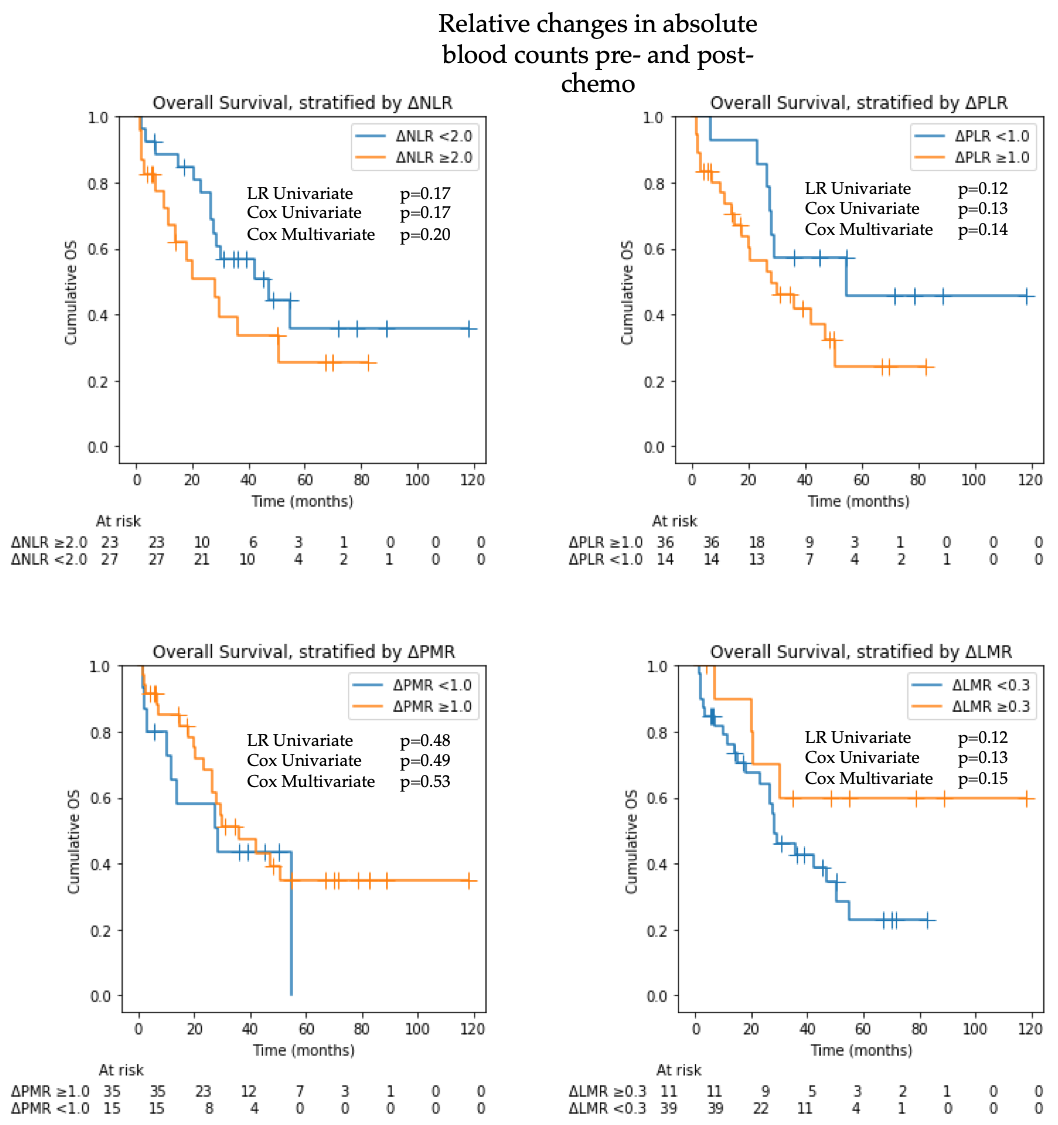

Supplement: Supplementary file 1 [file cancers-14-01818-s001.zip › supp fig 5.png]

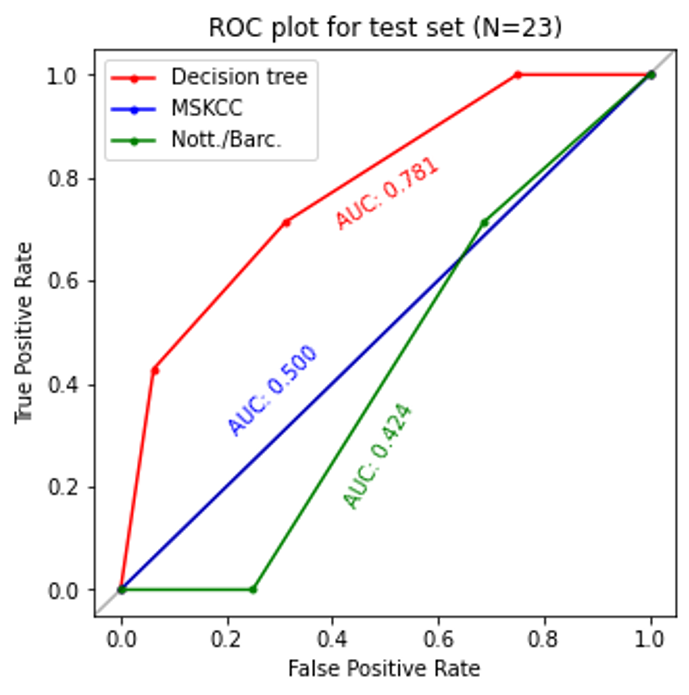

Supplement: Supplementary file 1 [file cancers-14-01818-s001.zip › supp fig 6.png]
